# Cultural Transmission Promotes the Emergence of Statistical Properties That Support Language Learning

**DOI:** 10.1111/cogs.70153

**Published:** 2025-12-28

**Authors:** Lucie Wolters, Simon Kirby, Inbal Arnon

**Affiliations:** ^1^ Department of Cognitive and Brain Sciences Hebrew University of Jerusalem; ^2^ Centre for Language Evolution, School of Philosophy, Psychology & Language Sciences University of Edinburgh; ^3^ Department of Psychology Hebrew University of Jerusalem

**Keywords:** Cultural transmission, Statistical structure, Zipfian distribution, Zipf's law of abbreviation, Learning

## Abstract

Language is passed across generations through cultural transmission. Prior experimental work, where participants reproduced sets of non‐linguistic sequences in transmission chains, shows that this process gives rise to two characteristic statistical properties of language that enhance its learnability: the statistical coherence of words and the Zipfian distribution of word frequencies. In this study, we extend this work in three ways. First, we replicate and strengthen previous findings using a browser‐based experimental procedure with a smaller dataset, demonstrating the robustness of these findings and creating a methodological platform for future research. Second, we show that learners are sensitive to the sequence information that emerges through cultural transmission by showing that reaction times are faster for higher transitional probabilities. These findings suggest that the learning of fine‐grained sequence information drives the emergence of statistically coherent units with a Zipfian frequency distribution. Third, we ask whether another cross‐linguistic property of language, Zipf's law of Abbreviation, emerges over cultural transmission. We find that the law is present in the sets produced by participants but that it does not evolve over transmission. We discuss how these findings support the proposal that production pressures alone may be sufficient to explain the consistently weak frequency–length correlation observed in natural language.

## 1 Introduction

Languages are repeatedly passed from one generation to the next via cultural transmission—the process by which a behavior is learned by observing it in others, who acquired it in the same way. A consequence of this type of transmission is that language evolves under a pressure to be learnable (Christiansen & Chater, 2008; Kirby, Cornish, & Smith, 2008). Over the past three decades, experimental and computational work simulating the process of cultural transmission has shown that it drives the evolution of increasingly learnable behaviors (e.g., Griffiths, Kalish, & Lewandowsky, 2008; Kirby et al., 2008, 2014) and gives rise to several core properties of language, such as compositionality and duality of patterning (Carr, Smith, Cornish, & Kirby, 2017; Kirby et al., 2008; Kirby & Tamariz, 2022; Verhoef, Kirby, & de Boer, 2014).

Recent work suggests that cultural transmission can also explain the emergence of two characteristic statistical properties of language. One, that words tend to be statistically coherent. This is often estimated by looking at the statistical relations between syllables, such as their transitional probabilities, which tend to be higher when both syllables belong to the same word, as opposed to when they cross a word‐boundary (Saksida, Langus, & Nespor, 2017; Stärk, Kidd, & Frost, 2022). And two, that word frequencies follow a Zipfian or near‐Zipfian distribution, where a few words occur frequently while many appear infrequently, with frequency declining according to a power law (Piantadosi, 2014; Zipf, 1949). This distribution has been observed across languages, linguistic modalities, speech registers, and different parts of speech (Bentz, Kiela, Hill, & Buttery, 2014; Ferrer i Cancho, 2005; Kaplan & Yu, 2025; Kimchi, Wolters, Stamp, & Arnon, 2023; Lavi‐Rotbain & Arnon, 2023; Mehri & Jamaati, 2017).

It has been proposed that cultural transmission drives the emergence of these properties because they facilitate language learning (Arnon & Kirby, 2024; Christiansen & Chater, 2016; Cornish, Dale, Kirby, & Christiansen, 2017; Shufaniya & Arnon, 2021). Across languages, the statistical coherence of words provides an informative cue to word segmentation, a fundamental challenge in language acquisition and a crucial part of language processing (Fourtassi, Börschinger, Johnson, & Dupoux, 2013; Gervain & Guevara Erra, 2012; Saksida et al., 2017). A large body of experimental work shows that adults, children, and young infants use these statistical patterns to discover word boundaries in continuous speech, demonstrating how the statistical coherence of words facilitates word segmentation (for review: Saffran & Kirkham, 2018). This finding is not limited to linguistic or auditory sequences and has also been found with non‐linguistic auditory stimuli and visual stimuli (Fiser & Aslin, 2002; Kirkham, Slemmer, & Johnson, 2002; Saffran, Johnson, Aslin, & Newport, 1999; Saffran & Kirkham, 2018). Segmentation is also facilitated by the Zipfian word frequency distribution of natural language: Infants, children, and adults are better at segmenting an artificial language when word frequencies follow a highly skewed distribution as opposed to a uniform one where all words appear equally often (Kurumada, Meylan, & Frank, 2013; Lavi‐Rotbain & Arnon, 2022; Wolters, Ota, & Arnon, 2025). Similar facilitative effects were found for visual statistical learning (Lavi‐Rotbain & Arnon, 2021), cross‐situational learning (Hendrickson & Perfors, 2019), word‐referent mapping (Wolters, Lavi‐Rotbain, & Arnon, 2024), and learning novel grammatical categories (Casenhiser & Goldberg, 2005; Wonnacott, Brown, & Nation, 2017).

Several experimental studies provide evidence for the emergence of these statistical properties through cultural transmission: The cultural transmission of sets of consonant strings was shown to increase their statistical coherence (Cornish et al., 2017), and repeated story transmission led to the emergence of a skewed frequency distribution of novel words (Shufaniya & Arnon, 2021). Moreover, recent work showed that the cultural transmission of non‐linguistic sequence sets drives the emergence of statistically coherent units with a skewed frequency distribution and that their emergence increases learnability. In this study, Arnon and Kirby (2024) reanalyzed data from an iterated sequence learning experiment where each participant reproduced a set of color sequences that was produced by a previous participant (Cornish, Smith, & Kirby, 2013). To test the emergence of statistical structure in the sequence sets, sequences were segmented where there were drops in the transitional probabilities of the colors, similar to how infants use drops in the transitional probabilities of syllables to segment speech (Saffran, Aslin, & Newport, 1996). Arnon and Kirby (2024) found that sets of initially random sequences evolved statistically coherent units (sub‐sequences) with a Zipfian frequency distribution. Importantly, sets with more skewed frequency distributions were learned better, suggesting that learnability pressures drive the emergence of these statistical properties.

The work by Arnon and Kirby (2024) showed that changes in the transitional probabilities of the sequence sets leads to the emergence of statistically coherent units with a Zipfian frequency distribution over transmission. This work has rested on the assumption that learners are sensitive to the transitional probabilities within the sequences and use these statistics during reproduction. However, there is no direct evidence that learners are sensitive to this cue. To investigate this, we ask whether learners are sensitive to the transitional probabilities that emerge through iterated learning, and in doing so, provide a better understanding of the kind of learning that drives the emergence of the statistical properties. One way to analyze learners’ sensitivity to statistical patterns is to look at their online behavior as they reproduce sequence information, specifically at their reaction times. If learners are sensitive to the statistics of the input, then this should be reflected in their reproduction patterns over transmission: We should find that learners’ reaction times are faster for more probable transitions (Siegelman, Bogaerts, Kronenfeld, & Frost, 2019, 2018).

As a second goal of this study, we ask whether another cross‐linguistic universal, Zipf's law of Abbreviation, also emerges through cultural transmission. Zipf's law of Abbreviation states that the frequency of a word is inversely correlated with the length of that word, which can be generalized as the tendency of more frequent words to be shorter (Strauss, Grzybek, & Altmann, 2007; Zipf, 1949). This correlation has been found in spoken and written language corpora across many languages (Bentz & Ferrer Cancho, 2016; Petrini et al., 2023; Piantadosi, Tily, & Gibson, 2011; Sigurd, Eeg‐Olofsson, & Van Weijer, 2004; Teahan, Wen, McNab, & Witten, 2000). Zipf's law of Abbreviation is typically explained to arise from an accuracy pressure from the listener—promoting longer words to reduce ambiguity—and an efficiency pressure from the speaker—promoting shorter words to reduce production effort. The trade‐off between these two pressures is argued to give rise to an inverse correlation between frequency and length (Kanwal, Smith, Culbertson, & Kirby, 2017; Piantadosi et al., 2011; Zipf, 1949), which is supported by experimental studies showing the emergence of the law in communicative contexts (Kanwal et al., 2017; Krauss & Weinheimer, 1964). However, recent work has challenged the necessity of communication for the emergence of the law (Morin & Koshevoy, 2024; Ueda & Washio, 2021). Specifically, it has been proposed that a pressure from the speaker to reduce production costs may be sufficient to account for its emergence, as speakers may selectively shorten frequent words during production (Morin & Koshevoy, 2024). Over time, this selective shortening will lead to the emergence of the law, even in the absence of listener‐related pressures. A recent agent‐based model even suggests that the law can arise when shortening is applied to all words, irrespective of their frequency: Since high frequency words are produced more often, they undergo shortening more frequently, resulting in a correlation between frequency and length consistent with Zipf's law of Abbreviation (Morin & Koshevoy, 2024). Using an iterated learning design where there are no communicative pressures, such as the sequence learning paradigm in Arnon and Kirby (2024), allows us to ask whether Zipf's law of Abbreviation emerges through cultural transmission when the learned signals are not used for communication. If a trade‐off between pressures for accuracy and efficiency is a necessary condition for the emergence of Zipf's law of Abbreviation, then we should not observe it in iterated learning contexts where signals are not used communicatively. On the other hand, if the law can arise purely through production‐based mechanisms, then we may expect to see it even in non‐communicative contexts.

Following Arnon and Kirby (2024), we will use an iterated sequence learning experiment in which participants reproduce sets of color sequences. Based on previous findings, we predict that sets of randomly generated sequences will evolve to have statistically coherent units, a skewed frequency distribution that approaches a Zipfian power law, and a positive correlation between distribution skew and transmission accuracy, suggesting that pressures from sequence learning contribute to the emergence of these statistical properties. In addition, we will ask whether the sequence information that emerges over transmission is reflected in the online behavior of learners by analyzing the reaction times with which learners reproduce the color sequences. We predict that more probable transitions will have faster reaction times. Last, we ask whether Zipf's law of Abbreviation will emerge over transmission, indicated by the emergence of a correlation between unit frequency and length.

## 2 Online iterated sequence learning experiment

This study was pre‐registered with the Open Science Foundation (osf.io/4rjeg).

### 2.1 Participants

A total of 146 participants participated in the study to collect data for ten transmission chains of ten generations. Participants were recruited on the crowdsourcing platform Prolific (prolific.com) and were pre‐screened to have English as their first language, to be between 18 and 40 years old, to have not participated in similar experiments run by the research team, and to have a prolific approval rate above 95%. Participants received monetary compensation at an hourly rate of £9. Informed consent was obtained from all participants.

During data collection, 46 participants were excluded based on the following pre‐registered exclusion criteria: (1) More than eight invalid trials in round two of the experiment (*n* = 28), (2) more than three incorrect trials during practice (*n* = 9), and (3) using recording devices to complete the study, based on their answer to a question posed at the end of the study (*n* = 9).^1^ Excluded participants were removed from the transmission chains and replaced with a new participant.

### 2.2 Procedure

**Fig. 1:**
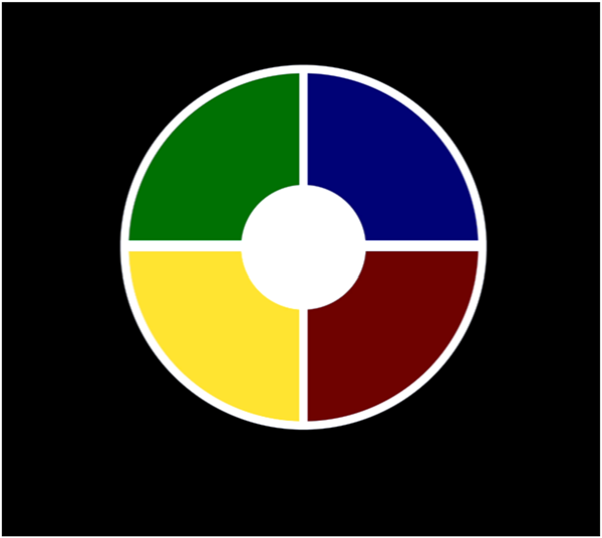
The playing board.

Participants saw sequences of colors (red, yellow, green, and blue) on a playing board and were asked to immediately reproduce them (using the design of the Simon Game, see Fig. 1). Each participant saw a set of 30 sequences, repeated in two rounds, with a self‐timed break in between. The set produced in the second round was transmitted to the next participant in the transmission chain. The presentation order of the 30 sequences was randomized for each round. The sequences for the initial generation of each chain were randomly generated and had a length of 12. Accordingly, the distribution of the individual colors was near‐uniform in all randomly generated sets.

At the start of the experiment, participants were told they would see and repeat 60 sequences (see the Appendix for full instructions). After seeing the playing board for 400 ms, they saw a sequence of color‐flashes.^2^ Each color lit up for 300 ms, with an interval of 300 ms between individual flashes. At the end of the sequence, the prompt “repeat, and press spacebar when you are done” appeared below the playing board. Participants reproduced the sequences using the q, w, a, and s keys on their keyboard, which, respectively, mapped onto the top‐left, top‐right, bottom‐left, and bottom‐right locations of the playing board.^3^ A color lit up for 300 ms when a key was pressed. After each trial, participants’ accuracy was displayed as a percentage score in the center of the playing board. This was calculated as the inverse Levenshtein edit distance^4^ between the seen and produced sequence, divided by the length of the longest sequence. Before starting the test trials, participants reproduced randomly generated practice sequences with a length of four. Participants continued to the test phase of the experiment when they reached the maximum of six training trials or when they correctly reproduced three sequences in a row. The experiment took 20–30 min to complete.

Trials were invalid when the length of the produced sequence fell outside the range of 8 to 16. This was done to ensure that participants did not adopt a strategy of only reproducing a few colors, and to keep learnability pressures from sequence length constant across generations. We still allowed for a range of sequence lengths so that we could detect potential trends in sequence shortening or lengthening. A trial was also invalid when a participant waited longer than five seconds to start reproducing the sequence. Invalid trials in the second round were repeated at the end, with a maximum of eight repeated trials. Participants that had more than eight invalid trials in the second round of the experiment were excluded and replaced by a new participant in the transmission chain.

## 3 Results

We analyzed the sequence sets that were reproduced in the second round of the experiment (the sets that were transmitted in the transmission chains). We used linear mixed‐effects regression models, implemented with the lme4 library (Bates et al., 2015) in R (R Core Team, 2021), for statistical analysis. All data and analyses can be accessed on the Open Science Foundation project page of this study: osf.io/6gu8z.

### 3.1 Sequence sets become easier to reproduce over generations

**Fig. 2:**
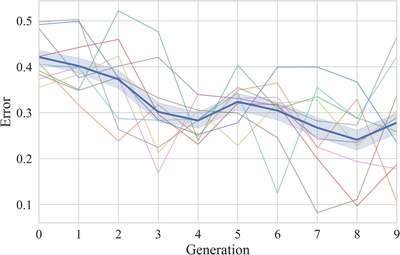
Transmission error in reproducing sequences decreases over transmission, suggesting that sequence sets become easier to reproduce over generations. The dark‐blue line with error bars represents the mean error of sequences across chains; error bars represent the 95% confidence intervals across sequences. The lines without error bars represent the mean transmission error of individual chains.

The transmission error for each trial was calculated as the normalized Levenshtein edit distance between the seen and produced sequence, which is calculated as the minimum number of insertions, deletions, and substitutions required to turn one sequence into the other, divided by the length of the longest sequence. Fig. 2 shows that transmission error decreases from 0.43 in generation zero (sd by chain = 0.05) to 0.28 in generation nine (sd by chain = 0.11), as expected if sets become more learnable. A model with error on trial as the outcome variable, a fixed effect of generation, and a by‐chain random slope for generation indicates that error decreases significantly over generations (β = −0.02, *SE* = 0.003, *t* = −6.43, *p* < .001). Sequence length also decreased over transmission, from an initial length of 12 in generation zero to 9.4 in generation nine (sd by chain = 0.7). Since shorter sequences are easier to reproduce, we included sequence length as a fixed effect in the model. Generation remains a significant predictor of error when controlling for sequence length (generation: β = −0.01, *SE* = 0.002, *t* = −5.13, *p* < .001, sequence length: β = 0.02, *SE* = 0.002, *t* = 9.3, *p* < .001). In addition, sequences were more likely to be reproduced correctly (error = 0) over transmission, increasing from 0% correct sequences in generation zero to 17% correct sequences in generation nine. A model with transmission success on trial (0 = incorrect, 1 = correct) as the outcome variable, fixed effects of generation and sequence length, and a by‐chain slope for generation confirms a significant increase in success over generations (generation: β = 0.17, *SE* = 0.05, *z* = 3.27, *p* < .01; sequence length: β = −0.57, *SE* = 0.06, *z* = −9.97, *p* < .001).^5^^,^ 6

These findings indicate that sets evolve to be easier to reproduce. This is a typical finding in iterated learning studies and shows how behaviors adapt to be increasingly learnable over transmission.

### 3.2 Segmentation method

**Fig. 3:**
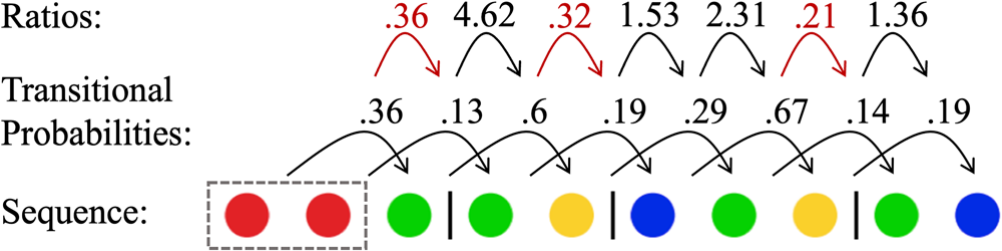
Visualization of the segmentation method developed by Arnon & Kirby (2024). Sequences were segmented based on the ratios of the transitional probabilities of colors. The figure shows the 11th sequence in the second generation of chain H. The ratios in red are below the segmentation threshold of 0.41. The black lines between the colors indicate the unit boundaries posited by the segmentation method based on the ratios of the transitional probabilities.

**Fig. 4:**
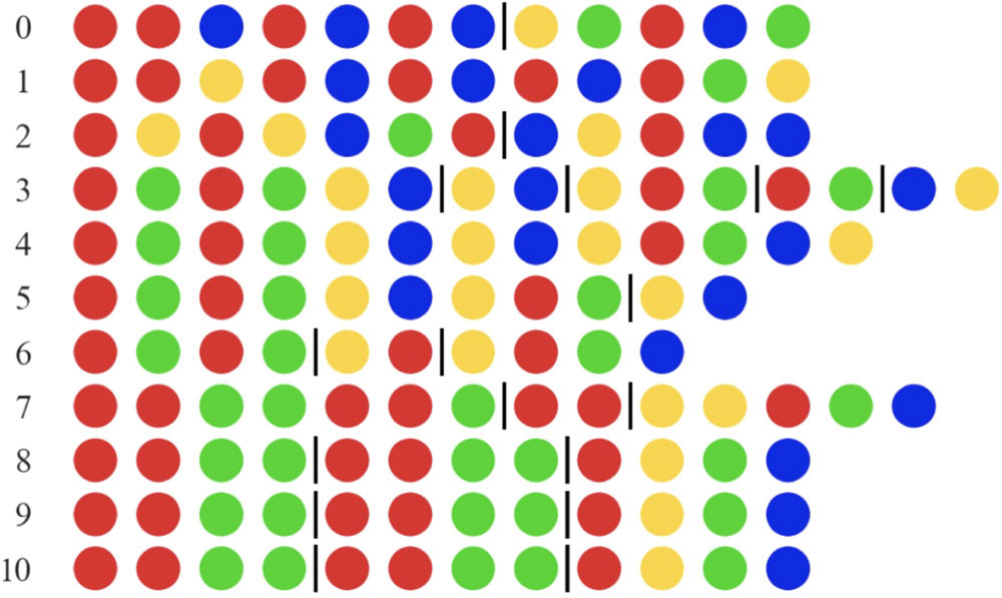
The change of a single sequence from chain D (out of a set of 30), over ten generations. The black lines between colors indicate the unit boundaries posited by the segmentation method (i.e., points in the sequence where the transitional probability ratio was lower than 0.41).

To analyze the statistical structure of the sequence sets, we segmented sequences into units using the segmentation method developed by Arnon and Kirby (2024). This method, which was motivated by the literature on statistical word segmentation, uses drops in the transitional probabilities between colors as indicators of unit boundaries, similar to how language learners can use drops in the transitional probabilities between syllables to discover word boundaries in continuous speech (Saffran et al., 1996). Transitional probabilities were calculated as the probability of a color to following the two preceding colors. The segmentation method identifies drops in transitional probability by measuring how much the transitional probability decreases from one transition to the next. It does this by calculating the ratio between two consecutive transitional probabilities. A non‐structured sequence set was used as a baseline to estimate when a drop in transitional probability is large enough to justify segmentation. To do this for the data of this study, we generated 500 random sequence sets of 30 sequences with a length of 12 (like the initial sets of the transmission chains). We then created an aggregated distribution of all the transitional probability ratios of these sets and took the lowest five percentile point of this distribution as the segmentation threshold (= 0.41). See Fig. 3 for a visualization of the segmentation method. Fig. 4 shows an example of the evolution of a single sequence over transmission.

### 3.3 Statistically coherent units emerge over transmission

**Fig. 5:**
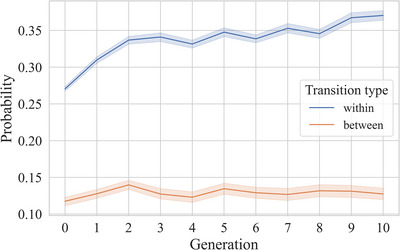
Mean probability of transitions within units and between units over generations. The mean probability of each transition type was calculated by dividing the sum of the probabilities of that transition type by the total count of those transitions. The error bars represent the 95% confidence intervals across individual probabilities. Over generations, the within‐unit transitional probabilities increase relative to the between‐unit transitional probabilities, indicating the emergence of statistically coherent units.

To analyze whether the statistical coherence of units increases over transmission, we compared the transitional probabilities of colors within units to those at the boundary between units. Fig. 5 shows that the within‐unit transitional probability increases over transmission, while the between‐unit transitional probability remains low. A model with transitional probability as the outcome variable, a fixed effect for transition type (within‐ or between‐unit), generation, and the interaction between them, and a random intercept for chain^7^ confirms that the probability of within‐unit transitions increases over transmission relative to between‐unit transitions (interaction between transition type and generation: β = 0.01, *SE* = 0.001, *t* = 8.54, *p* < .001). This means that segmented units become more statistically coherent over transmission, making the boundaries of the segmented units increasingly salient.

### 3.4 Frequency distributions become more Zipfian over generations

**Fig. 6:**
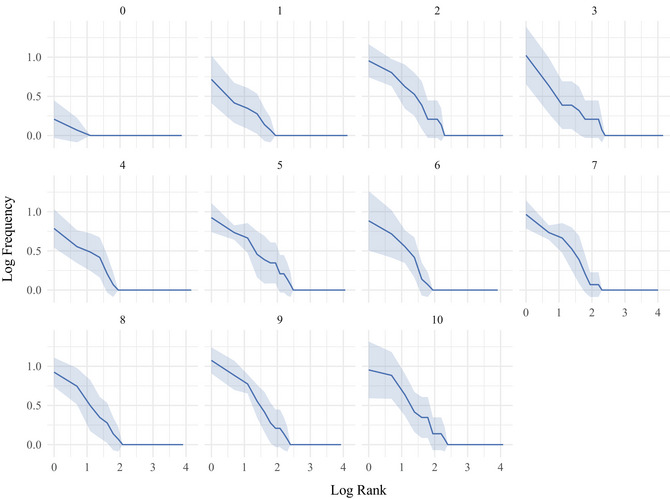
Skewed distributions emerge over generations. The frequency distribution of units with log frequency on the y‐axis and log rank on the x‐axis. Frequency represents the mean frequency of the chains, and error bars represent the 95% confidence intervals across chains.

**Fig. 7:**
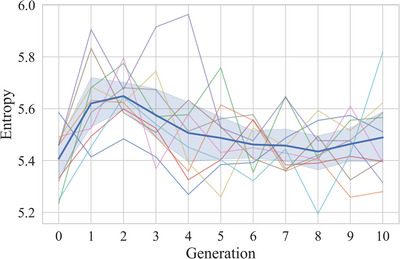
Entropy decreases over transmission. The dark‐blue line with error bars represents the mean entropy of chains; error bars represent the 95% confidence intervals across chains. The lines without error bars each represent the mean entropy of individual chains.

Fig. 6 shows that the frequency distributions of segmented units become more skewed over transmission. To quantify the structure of the frequency distributions, we looked at their entropy (Shannon, 1948). Entropy increases with set size (the number of unique units in the distribution) and decreases with distribution skew. Fig. 7 shows that after an initial increase, entropy decreases from 5.62 bits in generation one (sd by chain = 0.16) to 5.49 bits in generation ten (sd by chain = 0.16). Excluding generation zero, a model with entropy per set as the outcome variable, fixed effects of set size and generation, and a by‐chain random slope for generation indicates that entropy decreases with generation (β = −0.01, *SE* = 0.002, *t* = −2.39, *p* < .05), and increases with set size (β = 0.03, *SE* = 0.001, *t* = 30.38, *p* < .001). Set size decreases over transmission from 51 in generation one (sd by chain = 3.3) to 48 in Generation ten (sd by chain = 4.8), suggesting that a reduction in the number of unique units contributes to the overall decrease in entropy. However, entropy also decreases because of an increase in the skew of the distribution, indicated by an increase of entropy over generations after accounting for set size in the model.

To test whether the emerging distribution skew increasingly resembles a Zipfian power law, we look at the *R* **^2^** fit of the distributions to a linear regression between frequency and rank on a log–log scale. If the distributions become increasingly Zipfian, then we expect *R* **^2^** to increase over transmission. A model with *R* **^2^** as the outcome variable, a fixed effect of generation and a by‐chain random slope for generation confirms that *R* **^2^** increases significantly from 0.09 in generation zero (sd by chain = 0.15) to 0.53 in generation ten (sd by chain = 0.22; β = 0.02, *SE* = 0.01, *t* = 2.56, *p* < .05), indicating the emergence of a power law over transmission.

### 3.5 Frequency distributions show a better fit to a power law than to an exponential and uniform distribution

**Fig. 8:**
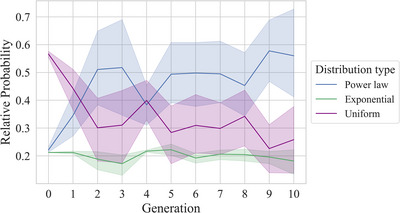
The mean relative probability of each distribution across generations. Error bars represent the 95% confidence intervals across chains. The relative probability of the power law model increases, eventually surpassing the alternatives. As expected, generation zero fits a uniform distribution best.

As some alternative distributions than the power law, such as the exponential and the log normal distribution, can also show an approximately linear relationship between frequency and rank on a log–log scale (Clauset, Shalizi, & Newman, 2009), we conducted additional analyses comparing the goodness‐of‐fit of the frequency distributions to the power law and several alternative distributions: the uniform and the exponential distribution. We included the uniform distribution to test whether the chains start out with a uniform frequency distribution (as would be expected since they were randomly generated and have not yet been shaped by learning) and gain a better fit to a power law over time. We included the exponential distribution since it can also appear approximately linear on a log–log scale (Clauset et al., 2009).^8^ To compare the fit of the observed frequency distribution to the three examined distributions (power law, uniform, exponential), we first estimated the parameters for each distribution using maximum likelihood estimation.^9^^,^ 10 ^,^ 11 Next, we derived the expected frequency distribution for each distribution based on the estimated parameters and the number of units and tokens in the observed distribution. We then quantified the goodness‐of‐fit of the observed distribution to the different examined distributions by calculating the Akaike Information Criterion (AIC). AIC is particularly useful here because it accounts for differences in model complexity (uniform = 0 parameters; power law = 1; exponential = 1). Following Sainburg, Theilman, Thielk, and Gentner (2019), we compared the AIC values for the different examined distributions by converting them into relative probabilities. This was done by subtracting each AIC from that of the best‐fitting model (ΔAIC), transforming the differences into relative likelihoods, and normalizing these likelihoods so they summed to one. If frequency distributions increasingly fit a power law over transmission, then we expect the relative probability of the power law model to increase over generations and eventually exceed that of the other two distributions. Fig. 8 shows that at generation zero, the uniform distribution provides the best fit. Importantly, over subsequent generations, the relative probability of the power law distribution increases, eventually surpassing the alternatives. These findings indicate that, over transmission, the frequency distributions gain a progressively better fit to a power law, compared to the uniform and exponential distribution.

### 3.6 The emergent statistical properties reflect the sequential ordering of colors

We wanted to rule out the possibility that the emergence of the Zipfian distribution is an artifact of the unigram distribution of colors or the length distribution of the sequences. Following Arnon et al. (2025), we tested to what extent a Zipfian frequency distribution emerges after shuffling the color tokens of the sequence set and redistributing them randomly across the sequences in the sets. This process results in a baseline that maintains the length of the sequences and the unigram distribution of the colors but destroys any sequential information in the original sets. If the emergence of the statistical properties reflects the sequential ordering of the color tokens, then we should find stronger evidence for the emergence of these properties in the original sets compared to the shuffled sets.

We shuffled the tokens of the sequence sets fifty times, creating fifty shuffled datasets for each original set. We then reran the segmentation method on each shuffled set. As before, we looked at the *R* **^2^** of their frequency distributions to a linear regression between frequency and length on a log–log scale. We fitted a model with *R* **^2^** per set as the outcome variable, with fixed effects of dataset (original, 100 sets; or shuffled, 5000 sets), generation and their interaction, and a by‐chain slope for generation. We find that the *R* **^2^** of the experimental sets is higher than that of the shuffled sets (β = 0.15, *SE* = 0.03, *t* = 4.37, *p* < .001) and that the *R* ^2^ increases more over generations in the experimental sets, compared to the shuffled sets, indicated by a significant interaction between dataset and generation (β = 0.02, *SE* = 0.00, *t* = 4.28, *p* < .001). This indicates that the emergence of the statistical properties reflects changes in the sequential ordering of the colors and is not a result of changes in the unigram distributions of colors or of the reduction in sequence length over generations.

To confirm that the statistical structure we observe is linked to the statistical cue used for sequence segmentation, we also created fifty “rotated” versions of each experimental set—again, following Arnon et al. (2025). For each rotation, the boundary locations were shifted forward by a randomly chosen number of positions (e.g., if boundaries were postulated at positions three and six in a sequence and the random number of rotations was two, those boundaries would now be at positions five and eight). Boundaries that moved past the end of a sequence were moved to the next sequence. We then looked at the frequency distribution of the units based on the rotated boundaries. The rotated baseline preserves the sequential order of the colors and the length distribution of the segmented units, but unit boundaries no longer align with drops in transitional probability. We fitted the same model as for the shuffled baseline and found that the *R* **^2^** of the experimental sets was significantly higher than that of the rotated sets (β = 0.08, *SE* = 0.03, *t* = 2.57, *p* < .05), and that the *R* **^2^** increases more over generations in the experimental sets, compared to the rotated sets, indicated by a marginally significant interaction between dataset and generation (β = 0.02, *SE* = 0.00, *t* = 4.28, *p* = .05).

**Fig. 9:**
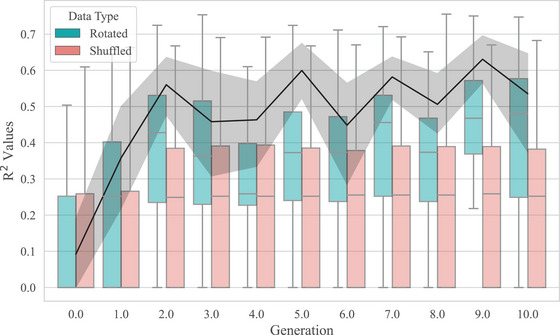
Comparison of the fit to a Zipfian distribution in the experimental data, compared to the shuffled and rotated datasets. The black line with error bars represents the mean *R* ^2^ of the experimental data; error bars represent the 95% confidence intervals across chains. The box plots represent *R* ^2^ values of the sets from the shuffled baseline (red) and the rotated baseline (blue).

Fig. 9 shows the *R* **^2^** of both baselines and the experimental data.

### 3.7 Sets with lower entropy are transmitted with fewer errors

**Fig. 10:**
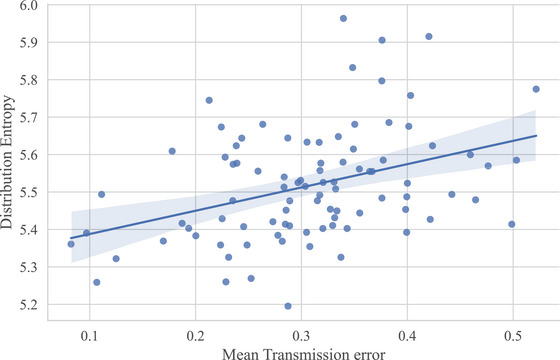
The correlation between the distribution entropy and the mean error of the sequence sets from generation one to nine. The figure shows that sets with lower entropy (i.e., that are more skewed) are easier to learn.

The previous analyses showed an increase in learnability over transmission and the emergence of statistical structure. Here, we want to ask whether sets with more statistical structure are indeed easier to reproduce by looking at the correlation between the distribution entropy and mean error of the sequence sets. The randomly generated sets of generation zero were excluded for this analysis. Fig. 10 shows an inverse correlation between the distribution entropy and mean transmission error of the sets, indicating that sets with lower entropy were easier to reproduce (*r*(88) = −0.38, *p* < .001). The correlation remains after normalizing entropy by set size,^12^ indicating that more skewed distributions are more learnable (*r*(88) = −0.21, *p* < .05). This suggests that the increase in the learnability over transmission is related to the increase of distribution skew over transmission.

As cultural transmission has been shown to reduce the complexity of transmitted behaviors (e.g., Kempe, Gauvrit, & Forsyth, 2015; Kirby, Tamariz, Cornish, & Smith, 2015), we wanted to rule out the possibility that our findings can be fully explained by such simplification of the sequence sets. To do this, we conducted additional analyses (Supporting Information C) examining: (1) the decrease in Kolmogorov complexity over generations (estimated using a standard compression algorithm); (2) the relationship between Kolmogorov complexity and the *R* ^2^ fit of the frequency distributions to a linear regression between frequency and rank on a log–log scale (the fit to a power law); and (3) how well complexity predicts transmission error compared to *R* ^2^. We find that our estimate of Kolmogorov complexity decreases over transmission, consistent with previous cultural transmission experiments, and that it is correlated with *R* ^2^. However, importantly, we also find that sequence sets with similar Kolmogorov complexity estimates can differ substantially in their statistical properties and that *R* ^2^ predicts transmission error better than complexity. These findings indicate that the emergence of the statistical properties cannot be attributed solely to a general loss of complexity over generations.

### 3.8 Reaction times reflect the emergence of statistical structure

Thus far, our results replicate the findings of Arnon and Kirby (2024) by showing the emergence of statistically coherent units that follow a Zipfian frequency distribution over transmission. These units reflect structure in the transitional probabilities within the sequences. Here, we ask if this emerging structure is reflected in participants’ online behavior. Specifically, we ask whether the reaction times of participants during reproduction—the time between consecutive keypresses for colors—is predicted by the probability of the transitions, such that reaction times are faster for more probable transitions. If so, this would tell us that learners are sensitive to the transitional probabilities we use to infer the statistical structure of the sequence sets.

All reaction times below 100 ms were excluded, to remove extremely short reaction times (0.3% of the data, 72 data points), as were all reaction times outside the range of 2 SD from the participant's mean, to minimize the effect of outliers (4.2% of the data, 1019 data points).^13^^,^ 14 The mean reaction time decreases from 456 ms in generation one (sd by chain = 63 ms) to 410 ms in generation ten (sd by chain = 84 ms). All analyses were conducted on log reaction times. To test our prediction, we used a model with log reaction time as the outcome variable. The model included fixed effects of generation (numerical, range = 1–10),^15^ position in the sequence (numerical, range = 3–16), and transitional probability (numerical, range: 0–1) and by‐chain random slopes for generation and a random intercept for color (categorical, four levels). The effect of transitional probability on reaction time was significant, with shorter reaction times for more probable transitions (β = −0.16, *SE* = 0.01, *t* = −10.37, *p* < .001), as expected if participants’ online behavior reflects the statistical structure of the sets^16^. There was also a negative effect of position (β = −0.01, *SE* = 0.0, *t* = −15.37, *p* < .001), with reaction time decreasing toward the end of sequences. Reaction time did not decrease significantly with generation (β = −0.01, *SE* = 0.01, *t* = −0.71, *p* = .5). These findings show that learners were sensitive to the transitional probabilities of colors, and with that the segmentation cue of the sequence sets.

### 3.9 Zipf's law of Abbreviation emerges but does not strengthen over transmission

**Fig. 11:**
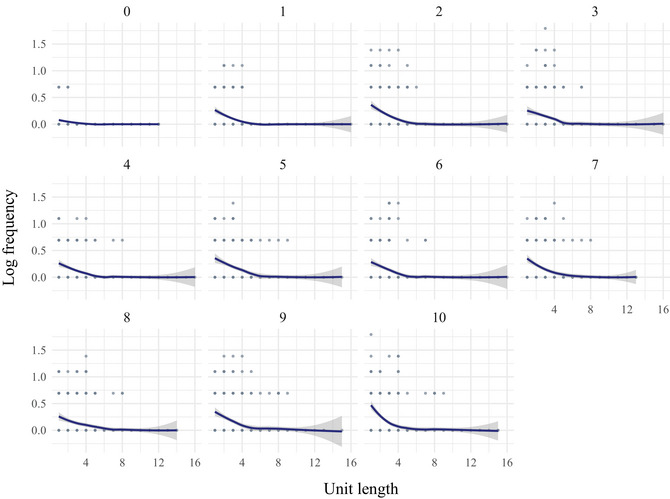
The frequency distribution of units over generations, with log frequency of units on the y‐axis, and unit length on the x‐axis.

We wanted to see whether Zipf's law of Abbreviation emerges through cultural transmission when the learned signals are not used for communication. Specifically, we look at whether unit frequency is predicted by unit length— such that short units tend to be more frequent —and whether this relationship strengthens over transmission. Fig. 11 shows the distribution of unit frequency across unit length over generations. To analyze this relationship, we fitted a Poisson regression model with frequency as the outcome variable and unit length, generation, and their interaction as fixed effects.^17^ We find that unit length significantly predicts frequency: shorter units tend to be more frequent (β = −0.03, *SE* = 0.00, *z* = −7.65, *p* < .001). However, the interaction between unit length and generation is not significant (β = −0.00, *SE* = 0.00, *z* = −1.94, *p* = .053), indicating that the strength of this relationship does not increase over generations. These results suggest that Zipf's law of Abbreviation, which describes an inverse relationship between unit frequency and length, is present in the sequence sets but does not become more pronounced over transmission.

To further examine whether the presence of the law is driven by pressures from production, we compared all the sets produced by participants (generations one to ten) to the randomly generated sets of generation zero. We refitted the model, this time excluding generation as a fixed effect and including a binary fixed effect indicating whether a set was participant‐produced or randomly generated, as well as its interaction with unit length. The model found a significant interaction (β = 0.03, *SE* = 0.01, *z* = 2.26, *p* < .05), indicating that the negative effect of unit length on frequency is stronger in the sets produced by participants compared to those that were randomly generated. Supporting this further, an analysis of generation zero alone suggests that unit length does not predict frequency in the randomly generated sets (β = −0.00, *SE* = 0.01, *z* = −0.35, *p* = .73), indicating that Zipf's law of Abbreviation is not present in the initial, randomly generated sequence sets but was introduced by the participants in their reproduction of the sets.

**Fig. 12:**
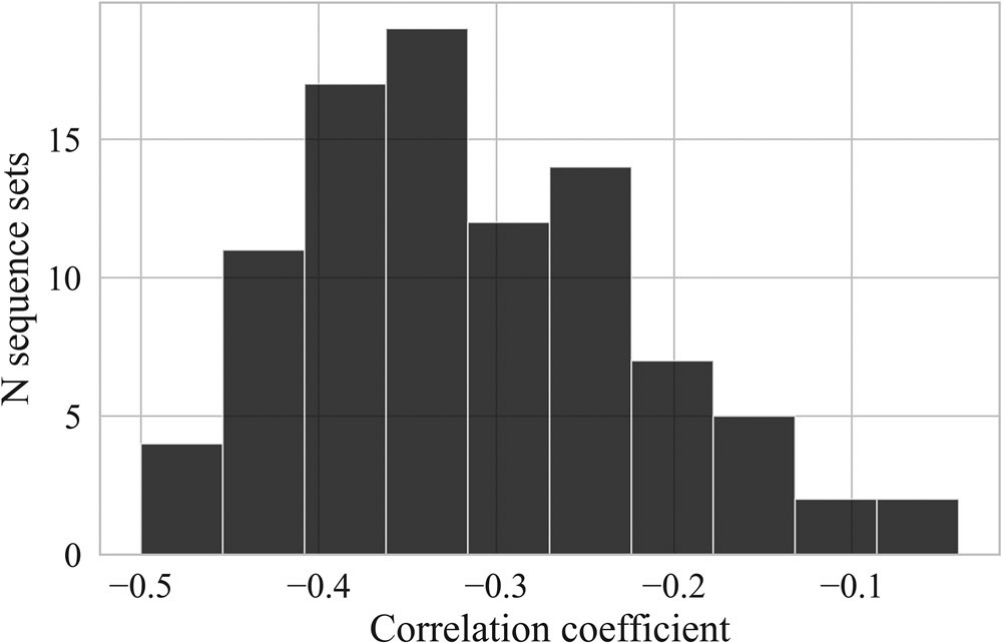
The distribution of Pearson's correlation coefficients in the sets produced by participants (generations 1–10). The correlations are relatively weak in the sets produced by participants.

Prior work has shown that when production pressures give rise to Zipf's law of Abbreviation, we should expect a characteristic pattern where highly frequent words tend to be short, while infrequent words may be either short or long—resulting in only a weak correlation between frequency and length (Morin & Koshevoy, 2024). Fig. 11 illustrates this pattern in our data as well: the less frequent a unit, the wider the range of its possible lengths. Consistent with this, Fig. 12 shows that the correlation between log unit frequency and length in the sequence sets produced by participants (generations 1–10) is relatively weak, with a mean Pearson correlation of –0.31.

To investigate whether the emergence of Zipf's law of Abbreviation is driven by the sequential ordering of the colors, we compared the data of generations 1–10 to the shuffled baseline described in Section 3.6. The full analyses are described in Supporting Information D. The findings suggest that the emergence of Zipf's law of abbreviation in the sets produced by participants is driven by changes in the sequential structure of the colors and is not solely a result of changes in the unigram distributions of colors or the reduction in sequence length.

## 4 Discussion

In this study, we investigated the role of cultural transmission in the emergence of several characteristic statistical properties of language. Replicating Arnon and Kirby (2024), we find that initially random sequence sets evolve to have statistically coherent units, characterized by increased within‐unit transitional probabilities, with a Zipfian frequency distribution. In addition, sets with lower distribution entropy were reproduced with less transmission errors, suggesting that the statistical properties emerge because they increase the learnability of the sets. Our results replicate those of Arnon and Kirby (2024) despite using smaller sequence sets (thirty sequences instead of sixty) and conducting the experiment on a browser‐based platform rather than in the lab. This demonstrates the robustness of the original findings and further supports the hypothesis that the learnability advantages of statistically coherent units and Zipfian frequency distributions contribute to their emergence in language. Moreover, as browser‐based experimental procedures are more cost‐effective and more time‐efficient, our study offers a scalable platform to study the cultural transmission of sequence information across larger and more diverse samples.

We conducted several analyses that strength the findings by Arnon and Kirby (2024). First, we compared the emergent frequency distributions to multiple alternative distributions beyond the power law. Previous work assessed the fit to a Zipfian distribution by looking at the *R* ^2^ fit of a linear regression between frequency and rank on a log–log scale, which is high for a power law distribution like the Zipfian. However, as shown by Clauset et al. (2009), high *R* ^2^ values are not unique to power laws; several alternative distributions can produce similarly strong linear relationships between frequency and rank on a log–log scale. We show that although the frequency distributions of the sequence sets initially best fit a uniform distribution, they better fit a power law over the course of cultural transmission, eventually outperforming the fit to a uniform and exponential distribution. Next, we asked whether the emergence of the statistical properties results from changes in the sequential structure of the sets rather than changes to the unigram frequency distribution of the colors or the reduction in sequence length. To do so, we compared the data to a baseline where we shuffled the color tokens across the sequences before segmentation. To additionally ensure that our findings are related to the specific cue we use for segmentation, we compared the data to a second baseline where we rotated the unit boundaries in the sequences so that segmentation no longer aligned with drops in transitional probabilities. The emergence of a Zipfian frequency distribution was significantly weaker in the two baselines compared to the experimental data, indicating that the emergence of the statistical properties results from changes to the sequential structure of the sequence sets and that the statistical properties we find are related to the specific cue we use to segment the sequences. Finally, because cultural transmission has been shown to reduce the complexity of transmitted behaviors (e.g., Kempe et al., 2015; Kirby et al., 2015), we wanted to rule out the possibility that our findings can be fully explained by such reductions in complexity. We found that while the estimated Kolmogorov complexity of sequence sets decreases over transmission, it does not fully explain the emergence of the statistical properties, or the increase in learnability. This suggests that the emergence of statistically coherent units with a Zipfian frequency distribution is not merely a by‐product of general simplification.

Previous work hypothesized that the emergence of the statistical properties reflects learners’ sensitivity to the sequence information of their input. In this study, we took a first step in testing this hypothesis by examining participants' online behavior during sequence reproduction. We found that reaction times were significantly faster for more probable color transitions, indicating that learners are sensitive to the sequence information that was used to analyze the statistical properties of the sets. Notably, this effect emerged despite sequences being presented as individual trials, suggesting that participants implicitly learned the set‐wide sequence information. With that, these findings contribute to the broader statistical learning literature, which typically relies on carefully curated sequences with categorical differences between within‐unit and between‐unit transitional probabilities (e.g., within‐unit transitional probabilities are often set to one, making units entirely predictable). Our findings show that much more fine‐grained statistical differences influence learners’ online behavior, enriching our understanding of the distributional information learners are sensitive to.

Extending previous findings, we asked whether cultural transmission drives the emergence of another linguistic universal, Zipf's law of Abbreviation. Our findings show that unit length significantly predicts unit frequency in the sequence sets produced by the participants (generations 1–10) but not in the randomly generated sets of generation zero. Moreover, we find that the strength of the frequency–length correlation did not increase over generations. Instead, Zipf's law of Abbreviation emerges early in transmission and then remains constant over generations. As the sequence reproduction task in this study did not involve any communication, these findings support the hypothesis that production pressures alone can drive the emergence of Zipf's law of Abbreviation (Morin & Koshevoy, 2024). Interestingly, the strength of the frequency–length correlation in generation one to nine (mean −0.31 when averaged across chains) is comparable to what is found in natural languages, where correlations range around −0.3 (see Fig. 2, p. 5, in Morin & Koshevoy, 2024). These consistently weak correlations reflect a key observation about Zipf's law of Abbreviation in language: While highly frequent units tend to be short, infrequent units may be either short or long. Prior work has suggested that both these features of Zipf's law of Abbreviation—a weak correlation and the presence of short infrequent units—are predicted when production pressures drive the law of Abbreviation (Morin & Koshevoy, 2024). This may also explain why repeated transmission does not further strengthen the correlation: A single round of reproduction may already sufficiently reduce production pressures, creating a ceiling effect.

Our findings suggest that learning pressures during cultural transmission give rise to characteristic statistical properties of language, but it is still an open question what precise learning mechanisms drive these outcomes. Prior work hypothesized that the emergence of the statistical properties arises from the repeated segmentation and learning of a set of units by each generation (Arnon & Kirby, 2024). Indeed, learners have been shown to break sequences into smaller, manageable units to facilitate the encoding and recall of sequential information (Bo & Seidler, 2009; Sakai, Kitaguchi, & Hikosaka, 2003; Verwey & Eikelboom, 2003). These findings are part of a broader literature showing that “chunking” is a fundamental mechanism of learning and memory, supporting skill acquisition across domains, including language (Arnon, 2021; Arnon, McCauley, & Christiansen, 2017; Chase & Simon, n.d.; Christiansen & Chater, 2016; Lieven, Pine, & Baldwin, 1997; Miller, 1956). Moreover, a recent computational model of iterated sequence learning suggests that segmentation will promote the emergence of statistically coherent units with a Zipfian frequency distribution (Wolters et al., 2025), consistent with the hypothesis that segmentation supports the emergence of these properties in language. Future research could test the role of segmentation in the emergence of these properties more directly using explicit measures of word segmentation, for example by comparing the recognition of sub‐sequences that vary in statistical coherence.

Finally, one feature of our study that stands out is that the sequences do not have any meaning. This design choice allowed us to isolate the effects of sequence learning from any biases from semantics or from language more broadly. Our findings suggest that the statistical structure of language may be driven by domain‐general learning pressures, rather than pressures that are specific to language. This creates interesting predictions about their emergence in other behaviors than language, specifically ones that do not have semantics. For example, music is another sequential behavior that is culturally transmitted but lacks language‐like semantics and, moreover, has been found to share certain statistical properties with language, such as Zipfian frequency distributions (Mehr et al., 2019; Perotti & Billoni, 2020). Similarly, we may expect to find similar statistical properties in the communication systems of other species that culturally transmit sequential signals for the purposes of sexual display or territorial defense rather than conveying complex meanings (e.g., several species of songbirds or whales). Supporting this hypothesis, recent work applied the infant‐inspired segmentation method developed by Arnon and Kirby (2024) to eight years of humpback whale song recordings and similarly found statistically coherent sub‐sequences that exhibited a Zipfian frequency distribution, as well as evidence for Zipf's law of Abbreviation (Arnon et al., 2025). Together with the study of the cultural transmission of human behavior, such research highlights the crucial role of learning and transmission in the emergence of structure and can help us better understand what processes an mechanisms are sufficient or necessary for the emergence of the characteristic statistical properties of human language, as well as the extent to which these are shared across different species.

## Supporting information



Supporting Information

## Notes

1 This exclusion rate is comparable to exclusion rates reported in other iterated learning studies that were conducted online (Keogh, Kirby, & Culbertson, 2024; Motamedi, Wolters, Naegeli, Kirby, & Schouwstra, 2022; Uddenberg, Thompson, Vlasceanu, Griffiths, & Todorov, 2023).

2 Hex color codes of the colors on the playing board: green: #005500, red: #550000, blue: #000055, yellow: #D5BE1A. Hex color codes of the colors when flashing: green: #00FF00, red: #FF0000, blue: #0000FF, yellow: #FFFC00.

3 At the start of the experiment, participants were asked to select whether they have a QWERTY or AZERTY keyboard. For participants with an AZERTY keyboard, the a, z, q, and s keys respectively mapped onto the top‐left‐, top‐right‐, bottom‐left‐ and bottom‐right‐location of the playing board.

4 The edit distance is the minimum number of insertions, deletions, and substitutions required to turn one sequence into the other.

5 The models in this section were also run with an added random intercept for item, which refers to each individual sequence in the sequence sets of generation zero, resulting in a variable with 300 levels. The main effects of generation and sequence length were still significant and have comparable effect sizes in these models. The full model results are included in the analysis script on the OSF page of this study.

6 The models in this section were also run with an added fixed effect for trial number. Trial number was not found to be a significant predictor in any of the described models and was therefore excluded as a fixed effect from all models. The full model results including a fixed effect for trial number are included in the analysis script on the OSF of this study.

7 The model was initially fitted with a by‐chain random slope for generation but it failed to converge due to singularity. The full model results are included in the analysis script on the OSF page of this study.

8 The log normal distribution also appears approximately linear on a log–log scale, but we chose not to include it in our analysis because log normal and power law fits are often statistically indistinguishable with a limited number of ranks as in our data. Furthermore, if we were to include the log normal distribution in our model comparison, using AIC as the criterion, it would be penalized for having two parameters, compared to the single parameter of the power law. Given that the fits are nearly identical on small datasets, the power law will almost always show a better fit because of this parameter penalty.

9 We implemented maximum likelihood estimation using SciPy's optimize.minimize function.

10 See Supporting Information A for the mathematical formulas for each distribution.

11 See Supporting Information B for an analysis of the estimated α parameters for the power law models over transmission.

12 Normalized entropy, also referred to as Efficiency, is calculated by subtracting the actual entropy from the maximum possible entropy for the given set size, and then dividing this difference by the maximum entropy. This results in a number between 0 and 1 that represents the fraction of the maximum possible entropy.

13 We only analyzed tokens for which both reaction times and transitional probabilities were available. Because the transitional probability of a token depends on the two preceding tokens, the first two tokens of each sequence lack transitional probability values, which accounts for 11% of the data (3000 tokens).

14 The analyses were also conducted without excluding any outliers, and the results did not change. These analyses are available in the analysis script on the study's OSF page.

15 Generation zero is not part of this dataset, as there is no reaction data for the randomly generated sequence sets.

16 In a separate model we added an interaction between generation and transitional probability to see whether transitional probabilities are more predictive of reaction times in later generations. The model revealed no significant interaction. This analysis is available in the analysis script on the study's OSF page.

17 We initially ran the model with a by‐chain random slope for generation, but the model did not converge with a random effect structure. These model results are included in the analysis script on the OSF page of this study.

## Experiment Instructions

**Table:**

Instructions, Screen 1:	“Welcome! In this study you will be playing a game. You will see a sequence of colours on the playing board displayed below. You will have to repeat the sequence by choosing the right colours in the order they were presented.”
Instructions, Screen 2:	*For participants with QWERTY keyboards*: “You can control the playing board by pressing ‘Q’, ‘W’, ‘A’, and ‘S’ on your keyboard. The key to colour mapping is displayed on the playing board below. **Try it out now!**” *For participants with AZERTY keyboards*: “You can control the playing board by pressing ‘A’, ‘Z’, ‘Q’, and ‘S’ on your keyboard. The key to colour mapping is displayed on the playing board below. **Try it out now!**”
Instructions, Screen 3:	ʻʻEach time you see a sequence of colours, repeat the sequence, and press the spacebar when you are done. To start a new sequence, press spacebar. We will start with some practice sequences. You can start the game after you correctly repeat 3 sequences in a row. Press ‘Next’ to start.ʼʼ
Instructions after practice trials:	ʻʻWell done! You are now ready to play the game. You will see and repeat 60 sequences. You will get the opportunity to take a break halfway. Note that the sequences will be longer than in the practice round. Try to repeat the whole sequence, even when you are not sure. Click ‘Next’ to start.
Break instructions:	“You can take a break now! When you are ready to continue with the game, press ‘Continue’ below.”
Recording question:	“Did you use any recording devices (e.g., pen and paper, screen recording, a camera) to complete the experiment? Please answer honestly. Your answer will not affect your approval rate on Prolific or your payment.”

## References

[cogs70153-bib-0001] Arnon, I. (2021). The Starting Big approach to language learning. Journal of Child Language, 48(5), 937–958. 10.1017/S0305000921000386 34219627

[cogs70153-bib-0002] Arnon, I. , & Kirby, S. (2024). Cultural evolution creates the statistical structure of language. Scientific Reports, 14(1), 5255. 38438558 10.1038/s41598-024-56152-9 PMC10912608

[cogs70153-bib-0003] Arnon, I. , Kirby, S. , Allen, J. A. , Garrigue, C. , Carroll, E. L. , & Garland, E. C. (2025). Whale song shows language‐like statistical structure. Science, 387(6734), 649–653. 10.1126/science.adq7055 39913578

[cogs70153-bib-0004] Arnon, I. , McCauley, S. M. , & Christiansen, M. H. (2017). Digging up the building blocks of language: Age‐of‐acquisition effects for multiword phrases. Journal of Memory and Language, 92, 265–280. 10.1016/j.jml.2016.07.004

[cogs70153-bib-0005] Bates, D. , Mächler, M. , Bolker, B. , & Walker, S. (2015). Fitting linear mixed‐effects models using lme4 . (arXiv:1406.5823). arXiv. 10.48550/arXiv.1406.5823

[cogs70153-bib-0006] Bentz, C. , & Ferrer Cancho, R. (2016). Zipf's law of abbreviation as a language universal. Proceedings of the Leiden Workshop on Capturing Phylogenetic Algorithms for Linguistics , Leiden, the Netherlands. 10.15496/publikation-10057

[cogs70153-bib-0007] Bentz, C. , Kiela, D. , Hill, F. , & Buttery, P. (2014). Zipf's law and the grammar of languages: A quantitative study of Old and Modern English parallel texts. Corpus Linguistics and Linguistic Theory, 10(2), 175–211. 10.1515/cllt-2014-0009

[cogs70153-bib-0008] Bo, J. , & Seidler, R. D. (2009). Visuospatial working memory capacity predicts the organization of acquired explicit motor sequences. Journal of Neurophysiology, 101(6), 3116–3125. 10.1152/jn.00006.2009 19357338 PMC2694099

[cogs70153-bib-0009] Carr, J. W. , Smith, K. , Cornish, H. , & Kirby, S. (2017). The cultural evolution of structured languages in an open‐ended, continuous world. Cognitive Science, 41(4), 892–923. 10.1111/cogs.12371 27061857 PMC5484388

[cogs70153-bib-0010] Casenhiser, D. , & Goldberg, A. E. (2005). Fast mapping between a phrasal form and meaning. Developmental Science, 8(6), 500–508. 10.1111/j.1467-7687.2005.00441.x 16246241

[cogs70153-bib-0011] Chase, W. G. , & Simon, H. A. (n.d.). The mind's eye in chess. In W. G. Chase (Ed.), Visualinformation processing (pp. 215–281). New York: Academic Press.

[cogs70153-bib-0012] Christiansen, M. H. , & Chater, N. (2008). Language as shaped by the brain. Behavioral and Brain Sciences, 31(5), 489–509. 18826669 10.1017/S0140525X08004998

[cogs70153-bib-0013] Christiansen, M. H. , & Chater, N. (2016). The Now‐or‐Never bottleneck: A fundamental constraint on language. Behavioral and Brain Sciences, 39, e62. 10.1017/S0140525X1500031X 25869618

[cogs70153-bib-0014] Clauset, A. , Shalizi, C. R. , & Newman, M. E. J. (2009). Power‐law distributions in empirical data. SIAM Review, 51(4), 661–703. 10.1137/070710111

[cogs70153-bib-0015] Cornish, H. , Dale, R. , Kirby, S. , & Christiansen, M. H. (2017). Sequence memory constraints give rise to language‐like structure through iterated learning. PLOS ONE, 12(1), e0168532. 10.1371/journal.pone.0168532 28118370 PMC5261806

[cogs70153-bib-0016] Cornish, H. , Smith, K. , & Kirby, S. (2013). Systems from sequences: An iterated learning account of the emergence of systematic structure in a non‐linguistic task. Proceedings of the Annual Meeting of the Cognitive Science Society , Berlin, Germany.

[cogs70153-bib-0017] Ferrer i Cancho, R. (2005). The variation of Zipf's law in human language. The European Physical Journal B—Condensed Matter and Complex Systems, 44, 249–257. 10.1140/epjb/e2005-00121-8

[cogs70153-bib-0018] Fiser, J. , & Aslin, R. N. (2002). Statistical learning of higher‐order temporal structure from visual shape sequences. Journal of Experimental Psychology: Learning, Memory, and Cognition, 28(3), 458–467. 10.1037/0278-7393.28.3.458 12018498

[cogs70153-bib-0019] Fourtassi, A. , Börschinger, B. , Johnson, M. , & Dupoux, E. (2013). Why is English so easy to segment? In V. Demberg & R. Levy (Eds.), Proceedings of the fourth annual workshop on cognitive modeling and computational linguistics (CMCL) (pp. 1–10). Stroudsburg, PA: Association for Computational Linguistics. https://aclanthology.org/W13‐2601

[cogs70153-bib-0020] Gervain, J. , & Guevara Erra, R. (2012). The statistical signature of morphosyntax: A study of Hungarian and Italian infant‐directed speech. Cognition, 125(2), 263–287. 10.1016/j.cognition.2012.06.010 22874070

[cogs70153-bib-0021] Griffiths, T. L. , Kalish, M. L. , & Lewandowsky, S. (2008). Theoretical and empirical evidence for the impact of inductive biases on cultural evolution. Philosophical Transactions of the Royal Society B: Biological Sciences, 363(1509), 3503–3514. 10.1098/rstb.2008.0146 PMC2607346 18801717

[cogs70153-bib-0022] Hendrickson, A. T. , & Perfors, A. (2019). Cross‐situational learning in a Zipfian environment. Cognition, 189, 11–22. 10.1016/j.cognition.2019.03.005 30903853

[cogs70153-bib-0023] Kanwal, J. , Smith, K. , Culbertson, J. , & Kirby, S. (2017). Zipf's Law of Abbreviation and the Principle of Least Effort: Language users optimise a miniature lexicon for efficient communication. Cognition, 165, 45–52. 10.1016/j.cognition.2017.05.001 28494263

[cogs70153-bib-0024] Kaplan, B. E. , & Yu, C. (2025). Near‐Zipfian distribution is prevalent in infant input. Proceedings of the Annual Meeting of the Cognitive Science Society , San Francisco, CA. https://escholarship.org/uc/item/1q63787s

[cogs70153-bib-0025] Kempe, V. , Gauvrit, N. , & Forsyth, D. (2015). Structure emerges faster during cultural transmission in children than in adults. Cognition, 136, 247–254. 10.1016/j.cognition.2014.11.038 25506774

[cogs70153-bib-0026] Keogh, A. , Kirby, S. , & Culbertson, J. (2024). Predictability and Variation in language are differentially affected by learning and production. Cognitive Science, 48(4), e13435. 10.1111/cogs.13435 38564253

[cogs70153-bib-0027] Kimchi, I. , Wolters, L. , Stamp, R. , & Arnon, I. (2023). Evidence of Zipfian distributions in three sign languages. Gesture, 22(2), 154–188. 10.1075/gest.23014.kim

[cogs70153-bib-0028] Kirby, S. , Cornish, H. , & Smith, K. (2008). Cumulative cultural evolution in the laboratory: An experimental approach to the origins of structure in human language. Proceedings of the National Academy of Sciences, 105(31), 10681–10686. 10.1073/pnas.0707835105 PMC2504810 18667697

[cogs70153-bib-0029] Kirby, S. , Griffiths, T. , & Smith, K. (2014). Iterated learning and the evolution of language. Current Opinion in Neurobiology, 28, 108–114. 10.1016/j.conb.2014.07.014 25062470

[cogs70153-bib-0030] Kirby, S. , & Tamariz, M. (2022). Cumulative cultural evolution, population structure and the origin of combinatoriality in human language. Philosophical Transactions of the Royal Society B: Biological Sciences, 377(1843), 20200319. 10.1098/rstb.2020.0319 PMC8666903 34894728

[cogs70153-bib-0031] Kirby, S. , Tamariz, M. , Cornish, H. , & Smith, K. (2015). Compression and communication in the cultural evolution of linguistic structure. Cognition, 141, 87–102. 10.1016/j.cognition.2015.03.016 25966840

[cogs70153-bib-0032] Kirkham, N. Z. , Slemmer, J. A. , & Johnson, S. P. (2002). Visual statistical learning in infancy: Evidence for a domain general learning mechanism. Cognition, 83(2), B35–B42. 10.1016/S0010-0277(02)00004-5 11869728

[cogs70153-bib-0033] Krauss, R. M. , & Weinheimer, S. (1964). Changes in reference phrases as a function of frequency of usage in social interaction: A preliminary study. Psychonomic Science, 1(1), 113–114. 10.3758/BF03342817

[cogs70153-bib-0034] Kurumada, C. , Meylan, S. C. , & Frank, M. C. (2013). Zipfian frequency distributions facilitate word segmentation in context. Cognition, 127(3), 439–453. 10.1016/j.cognition.2013.02.002 23558340

[cogs70153-bib-0035] Lavi‐Rotbain, O. , & Arnon, I. (2021). Visual statistical learning is facilitated in Zipfian distributions. Cognition, 206, 104492. 10.1016/j.cognition.2020.104492 33157380

[cogs70153-bib-0036] Lavi‐Rotbain, O. , & Arnon, I. (2022). The learnability consequences of Zipfian distributions in language. Cognition, 223, 105038. 10.1016/j.cognition.2022.105038 35123219

[cogs70153-bib-0037] Lavi‐Rotbain, O. , & Arnon, I. (2023). Zipfian distributions in child‐directed speech. Open Mind, 7, 1–30. 36891353 10.1162/opmi_a_00070 PMC9987348

[cogs70153-bib-0038] Lieven, E. V. M. , Pine, J. M. , & Baldwin, G. (1997). Lexically‐based learning and early grammatical development. Journal of Child Language, 24(1), 187–219. 10.1017/S0305000996002930 9154014

[cogs70153-bib-0039] Mehr, S. A. , Singh, M. , Knox, D. , Ketter, D. M. , Pickens‐Jones, D. , Atwood, S. , Lucas, C. , Jacoby, N. , Egner, A. A. , Hopkins, E. J. , Howard, R. M. , Hartshorne, J. K. , Jennings, M. V. , Simson, J. , Bainbridge, C. M. , Pinker, S. , O'Donnell, T. J. , Krasnow, M. M. , & Glowacki, L. (2019). Universality and diversity in human song. Science, 366(6468), eaax0868. 10.1126/science.aax0868 31753969 PMC7001657

[cogs70153-bib-0040] Mehri, A. , & Jamaati, M. (2017). Variation of Zipf's exponent in one hundred live languages: A study of the Holy Bible translations. Physics Letters A, 381(31), 2470–2477. 10.1016/j.physleta.2017.05.061

[cogs70153-bib-0041] Miller, G. A. (1956). Information and memory. Scientific American, 195(2), 42–47.

[cogs70153-bib-0042] Morin, O. , & Koshevoy, A. (2024). A cultural evolutionary model for the law of abbreviation. Topics in Cognitive Science. 10.1111/tops.12782 39718966

[cogs70153-bib-0043] Motamedi, Y. , Wolters, L. , Naegeli, D. , Kirby, S. , & Schouwstra, M. (2022). From improvisation to learning: How naturalness and systematicity shape language evolution. Cognition, 228, 105206. 10.1016/j.cognition.2022.105206 35810511

[cogs70153-bib-0044] Perotti, J. I. , & Billoni, O. V. (2020). On the emergence of Zipf ’s law in music. Physica A: Statistical Mechanics and Its Applications, 549, 124309. 10.1016/j.physa.2020.124309

[cogs70153-bib-0045] Petrini, S. , Casas‐i‐Muñoz, A. , Cluet‐i‐Martinell, J. , Wang, M. , Bentz, C. , & Ferrer‐i‐Cancho, R. (2023). The optimality of word lengths. Theoretical foundations and an empirical study . (arXiv:2208.10384). arXiv. 10.48550/arXiv.2208.10384

[cogs70153-bib-0046] Piantadosi, S. T. (2014). Zipf's word frequency law in natural language: A critical review and future directions. Psychonomic Bulletin & Review, 2(5), 1112–1130. 10.3758/s13423-014-0585-6 PMC4176592 24664880

[cogs70153-bib-0047] Piantadosi, S. T. , Tily, H. , & Gibson, E. (2011). Word lengths are optimized for efficient communication. Proceedings of the National Academy of Sciences , 108(9), 3526–3529. 10.1073/pnas.1012551108 PMC3048148 21278332

[cogs70153-bib-0048] R Core Team . (2021). R: A Language and environment for statistical computing [Computer software]. R Foundation for Statistical Computing. https://www.R‐project.org/

[cogs70153-bib-0049] Saffran, J. R. , Aslin, R. N. , & Newport, E. L. (1996). Statistical learning by 8‐month‐old infants. Science, 274(5294), 1926–1928. 10.1126/science.274.5294.1926 8943209

[cogs70153-bib-0050] Saffran, J. R. , Johnson, E. K. , Aslin, R. N. , & Newport, E. L. (1999). Statistical learning of tone sequences by human infants and adults. Cognition, 70(1), 27–52. 10.1016/S0010-0277(98)00075-4 10193055

[cogs70153-bib-0051] Saffran, J. R. , & Kirkham, N. Z. (2018). Infant Statistical Learning. Annual Review of Psychology, 69(1), 181–203. 10.1146/annurev-psych-122216-011805 PMC5754249 28793812

[cogs70153-bib-0052] Sainburg, T. , Theilman, B. , Thielk, M. , & Gentner, T. Q. (2019). Parallels in the sequential organization of birdsong and human speech. Nature Communications, 10(1), 3636. 10.1038/s41467-019-11605-y PMC6690877 31406118

[cogs70153-bib-0053] Sakai, K. , Kitaguchi, K. , & Hikosaka, O. (2003). Chunking during human visuomotor sequence learning. Experimental Brain Research, 152(2), 229–242. 10.1007/s00221-003-1548-8 12879170

[cogs70153-bib-0054] Saksida, A. , Langus, A. , & Nespor, M. (2017). Co‐occurrence statistics as a language‐dependent cue for speech segmentation. Developmental Science, 20(3), e12390. 10.1111/desc.12390 27146310

[cogs70153-bib-0055] Shannon, C. E. (1948). A mathematical theory of communication. The Bell System Technical Journal, 27(3), 379–423. 10.1002/j.1538-7305.1948.tb01338.x

[cogs70153-bib-0056] Shufaniya, A. , & Arnon, I. (2021). A cognitive bias for Zipfian distributions? Uniform distributions become more skewed via cultural transmission. Proceedings of the Annual Meeting of the 43th Cognitive Science Society , Virtual. https://escholarship.org/uc/item/66c40451

[cogs70153-bib-0057] Siegelman, N. , Bogaerts, L. , Armstrong, B. C. , & Frost, R. (2019). What exactly is learned in visual statistical learning? Insights from Bayesian modeling. Cognition, 192, 104002. 10.1016/j.cognition.2019.06.014 31228679

[cogs70153-bib-0058] Siegelman, N. , Bogaerts, L. , Kronenfeld, O. , & Frost, R. (2018). Redefining “learning” in statistical learning: What does an online measure reveal about the assimilation of visual regularities? Cognitive Science, 42(S3), 692–727. 10.1111/cogs.12556 28986971 PMC5889756

[cogs70153-bib-0059] Sigurd, B. , Eeg‐Olofsson, M. , & Van Weijer, J. (2004). Word length, sentence length and frequency—Zipf revisited. Studia Linguistica, 58(1), 37–52. 10.1111/j.0039-3193.2004.00109.x

[cogs70153-bib-0060] Stärk, K. , Kidd, E. , & Frost, R. L. A. (2022). Word segmentation cues in German child‐directed speech: A corpus analysis. Language and Speech, 65(1), 3–27. 10.1177/0023830920979016 33517856 PMC8886305

[cogs70153-bib-0061] Strauss, U. , Grzybek, P. , & Altmann, G. (2007). Word length and word frequency. In P. Grzybek (Ed.), Contributions to the science of text and language: Word length studies and related issues (pp. 277–294). Dordrecht, the Netherlands: Springer. 10.1007/978-1-4020-4068-9_13

[cogs70153-bib-0062] Teahan, W. J. , Wen, Y. , McNab, R. , & Witten, I. H. (2000). A compression‐based algorithm for Chinese word segmentation. Computational Linguistics, 26(3), 375–393. 10.1162/089120100561746

[cogs70153-bib-0063] Uddenberg, S. , Thompson, B. D. , Vlasceanu, M. , Griffiths, T. L. , & Todorov, A. (2023). Iterated learning reveals stereotypes of facial trustworthiness that propagate in the absence of evidence. Cognition, 237, 105452. 10.1016/j.cognition.2023.105452 37054490

[cogs70153-bib-0064] Ueda, R. , & Washio, K. (2021). On the relationship between Zipf`s law of abbreviation and interfering noise in emergent languages. In J. Kabbara , H. Lin , A. Paullada , & J. Vamvas (Eds.), Proceedings of the 59th annual meeting of the Association for Computational Linguistics and the 11th international joint conference on natural language processing: Student research workshop (pp. 60–70). Stroudsburg, PA: Association for Computational Linguistics. 10.18653/v1/2021.acl-srw.6

[cogs70153-bib-0065] Verhoef, T. , Kirby, S. , & de Boer, B. (2014). Emergence of combinatorial structure and economy through iterated learning with continuous acoustic signals. Journal of Phonetics, 43, 57–68.

[cogs70153-bib-0066] Verwey, W. B. , & Eikelboom, T. (2003). Evidence for lasting sequence segmentation in the discrete sequence‐production task. Journal of Motor Behavior, 35(2), 171–182. 10.1080/00222890309602131 12711587

[cogs70153-bib-0067] Wolters, L. , Lavi‐Rotbain, O. , & Arnon, I. (2024). Zipfian distributions facilitate children's learning of novel word‐referent mappings. Cognition, 253, 105932. 10.1016/j.cognition.2024.105932 39217784

[cogs70153-bib-0068] Wolters, L. , Ota, M. , & Arnon, I. (2025). Skewed distributions facilitate infants’ word segmentation. Cognition, 263, 106221. 10.1016/j.cognition.2025.106221 40532291

[cogs70153-bib-0069] Wonnacott, E. , Brown, H. , & Nation, K. (2017). Skewing the evidence: The effect of input structure on child and adult learning of lexically based patterns in an artificial language. Journal of Memory and Language, 95, 36–48. 10.1016/j.jml.2017.01.005

[cogs70153-bib-0070] Zipf, G. K. (1949). Human behavior and the principle of least effort: an introduction to human ecology. Cambridge, MA: Addison‐Wesley Press.

