# Supplementary material for: Cultural Transmission Promotes the Emergence of Statistical Properties That Support Language Learning
Source: Cogn Sci. 2025 Dec 28;49(12):e70153. doi: 10.1111/cogs.70153 (PMC12745064; doi:10.1111/cogs.70153)
Supplement: Supplementary file 1 — Supporting Information [file COGS-49-e70153-s001.pdf]

Supporting Information A: Distribution models

In Section 3.5 of the manuscript, we compare the unit frequency distribution of each sequence set to the three distribution fits: power-law, exponential, and uniform. The mathematical formulas for each distribution are given below. The denominator of all three models is the normalization constant, which ensures the probabilities sum to one. Figure 1 shows a visualization of the three models for a distribution with fifty ranks.

Power law distribution

The power law distribution assigns higher probability to small ranks (r) (1 = most frequent rank, …, N = least frequent rank), resulting in long tail with low probabilities. The scaling exponent $\alpha > 0$ controls how steeply the probabilities decay, which is the only free parameter of the distribution:

$$P(r \mid \alpha) = \frac{r^{-\alpha}}{\sum_{k=1}^{N} k^{-\alpha}}$$

Uniform distribution

The uniform distribution assumes that all ranks are equally likely, with N denoting the total number of ranks. The uniform distribution has no free parameters:

$$P(r) = \frac{1}{N}$$

Exponential distribution

The exponential distribution assigns probabilities that decay exponentially as a function of rank (r), with the rate of decay set by $\lambda > 0$, which is the only free parameter of the distribution:

$$P(r \mid \lambda) = \frac{e^{-\lambda r}}{\sum_{k=1}^{N} e^{-\lambda k}}$$

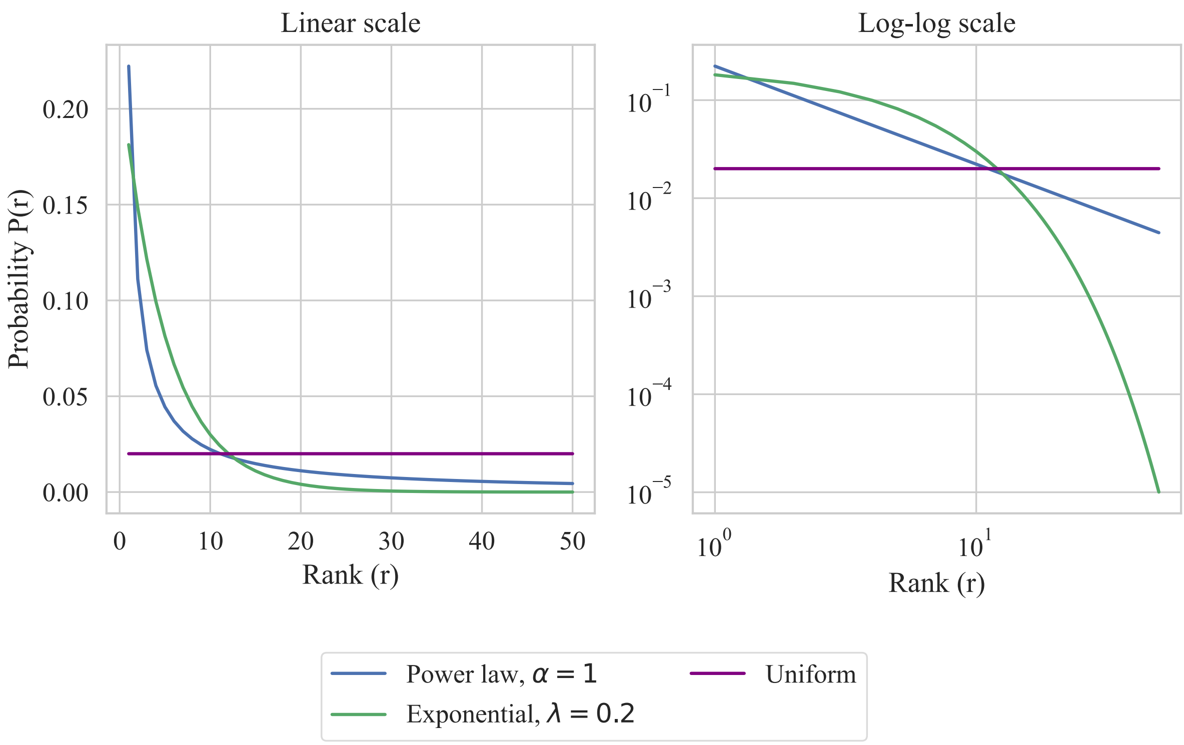

Figure 1: Visualization of the three distribution models for a frequency distribution with fifty ranks.

Supporting Information B: Estimated α parameter for the power law distribution

We estimated the α for the frequency distributions when fitted to a power law using maximum likelihood estimation, as described in Section 3.5 of the manuscript. Figure 1 shows alpha increases from 0.1 in generation zero to 0.28 in generation 10. A model with alpha value as the outcome variable, a fixed effect of generation, and a random intercept for chain indicates that the alpha values significantly increase over generations (**β** = 0.01, SE = 0.003, $t = 4.81$, $p < .001$)[1,2].

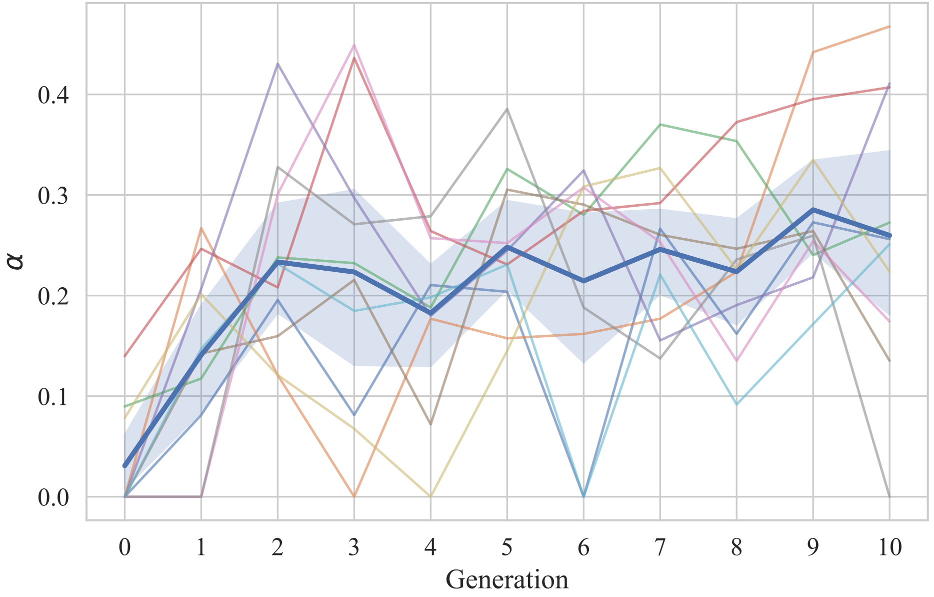

Figure 1: The estimated α parameter for a power law model increases over generations. The dark-blue line with error bars represents the mean α parameter of chains; error-bars represent the 95% confidence intervals across chains. The lines without error bars each represent the estimated α parameter of individual chains.

[1] The model was initially fitted with a by-chain random slope for generation; however, it failed to converge due to singularity. The random slope was removed for model convergence.

[2] We repeated the analysis after removing the randomly generated sequence sets from generation zero. Excluding generation zero did not lead to significant changes in the findings.

Supporting Information C: Estimating Kolmogorov complexity

Cultural transmission tends to decrease the complexity of transmitted systems. This is typically investigated by estimating the Kolmogorov complexity of behaviours or systems, which quantifies the complexity of a system by the length of its shortest possible description (given all possible algorithmic descriptions). With the following analyses we investigate whether (1) the complexity of the sequence sets decreases over cultural transmission, and (2) to what extent the emergence the statistical properties analysed in our study– statistically coherent units with a Zipfian frequency distribution– can be explained by a reduction in the complexity of the sequence sets, and (3) explain the learnability of the sets.

We estimated the Kolmogorev complexity of a sequence set by calculating the compression ratio of the sequence sets using the Zlib library (www.zlib.net), which was the ratio of the file size before and after compression (lower ratios indicating less complex sets). Using a linear mixed effects model with compression ratio as the dependent variable, a fixed effect of generation, and by-chain random slope for generation, we find that the complexity of sequence sets significantly decreases over generations ($\boldsymbol{\beta}$ = -0.001, SE = 0.00, t = -3.67, p < .01), from 0.43 in generation zero to 0.41 in generation ten (see Figure 1). We also found that complexity is significantly correlated with the R^2 fit of the frequency distribution to a linear regression between frequency and rank on a log-log scale (r(98) = 0.28, p = .01) but that the two measures are not interchangeable: sets with identical compression ratios differ widely in their statistical structure (e.g., for the six sets with a compression ratio of 0.43, distributions ranged from uniform to highly skewed with an R^2 of 0.76) (see figure 2).

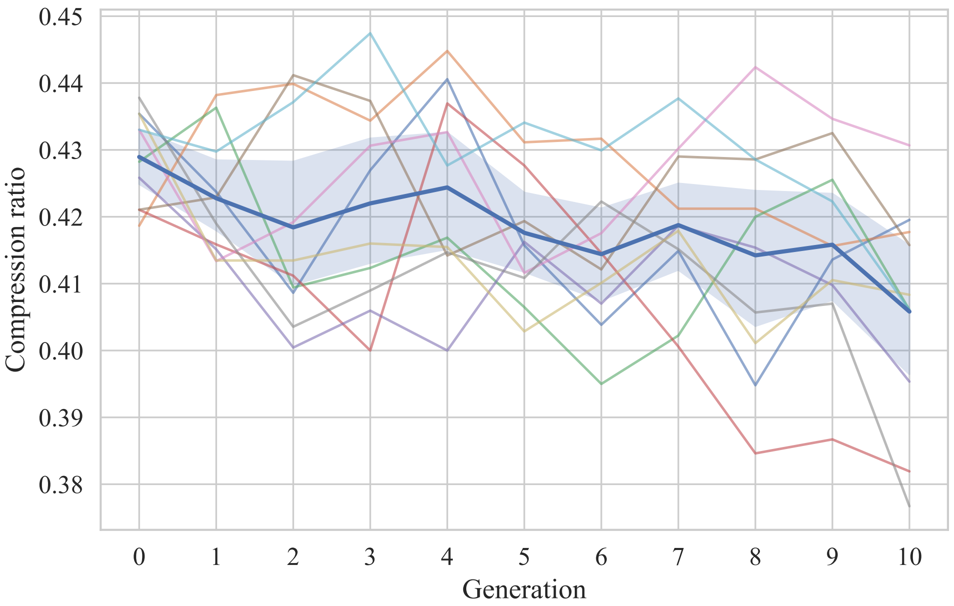

Figure 1: Sequence sets become less complex over generations, as indicated by a decrease in the compression ratio of sequence sets over time. The dark-blue line with error bars represents the mean complexity of sequence sets across chains; error-bars represent the 95% confidence intervals across sets. The colored lines without error bars represent the complexity over generations of individual chains.

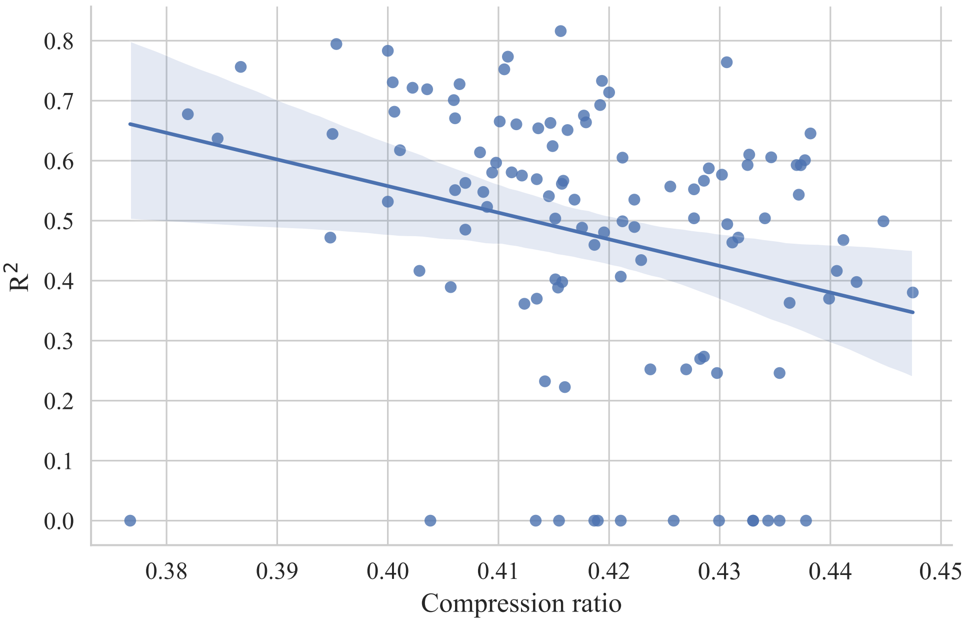

Figure 2: The correlation between the complexity of sequence sets and the fit of their unit frequency distribution to a power-law (R^2).

Next, we ask how well each measure (compression ratio and R^2) explains the decrease in transmission error over generations. We found that transmission error is not correlated with compression ratio (r(98) = 0.009, p = .93), but it is significantly correlated with the R^2 (r(98) = 0.27, $p < .01$), and with distribution entropy (entropy: r(98) = 0.25, $p < .01$; normalized entropy: r(98) = 0.32, $p < .01$). A set of ANOVA model comparisons confirmed that a model with R^2 and compression ratio as predictors has more explanatory power than a model with only compression ratio as its predictor ($F(1,97) = 13.57$, $p < 0.001$), but that compression ratio provides no additional explanatory power beyond power law fit alone ($F(1,97) = 1.21$, $p = 0.28$)[3].

Together, these analyses show that complexity decreases over transmission, consistent with previous cultural transmission studies; complexity is related to the statistical properties we investigate; but that equally complex sets can differ substantially in their statistical properties, and that the power law fit of the unit frequency distributions (R^2) is a better predictor of transmission error than overall complexity.

---

[3]The initial ANOVA model comparison was conducted on models that included a random-effects structure (by-chain random slope for generation). However, because two of the three models were overfitted due to low variance in the random effects, we repeated the analysis without random effects. The ANOVA comparisons with random effects are available in the analysis script on the OSF page.

Supporting Information D: Shuffled baseline for Zipf's law of Abbreviation

We tested whether the predictive relationship between frequency and length is stronger in the experimental sets of generation 1-10, compared to the shuffled sets (Section 3.6 in the main text). To do so, we fitted a Poisson regression model with frequency as the outcome variable, and unit length and data set (original, 100 sets; or shuffled, 5000 sets) and their interaction as fixed effects. We find that unit length is a better predictor of frequency in the experimental sets compared to the shuffled sets, indicated by a significant interaction between unit length and data set ($\boldsymbol{\beta}$ = 1.02, SE = 0.00, t = 4.76, p < .001). We do not find a significant interaction when comparing the experimental sets and shuffled sets of generation zero ($\boldsymbol{\beta}$ = 1.0, SE = 0.01, t = -0.23, p = .82). This indicates that the emergence of Zipf's law of abbreviation in the sets produced by participants is driven by changes in the sequential structure of the colours and is not solely a result of changes in the unigram distributions of colours or the reduction in sequence length. In addition, we also inspected the correlation coefficients for generations 1–10 after randomly shuffling the sequences. The shuffled sequence sets show a similar range to the experimental data (shuffled: –0.74 to 0.05; experimental: –0.50 to -0.04), with a slightly lower mean of –0.3 compared to –0.31 in the experimental data.
